# Supplementary figures and images for: Overexpression of ITGB3 in Peripheral Blood Mononuclear Cells of Relapsing-Remitting Multiple Sclerosis Patients
Source: Int J Mol Sci. 2025 Dec 16;26(24):12094. doi: 10.3390/ijms262412094 (PMC12733047; doi:10.3390/ijms262412094)

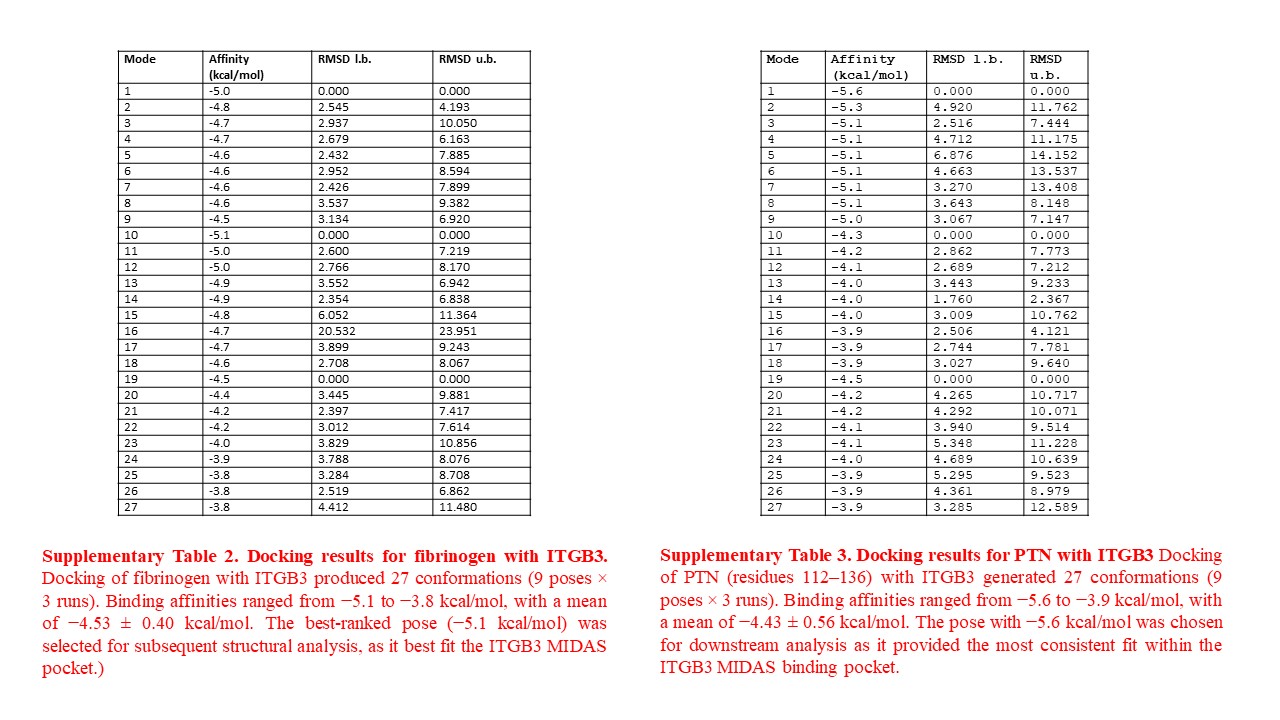

Supplement: Supplementary file 1 [file ijms-26-12094-s001.zip › Supp TAbles 2 and 3.JPG]

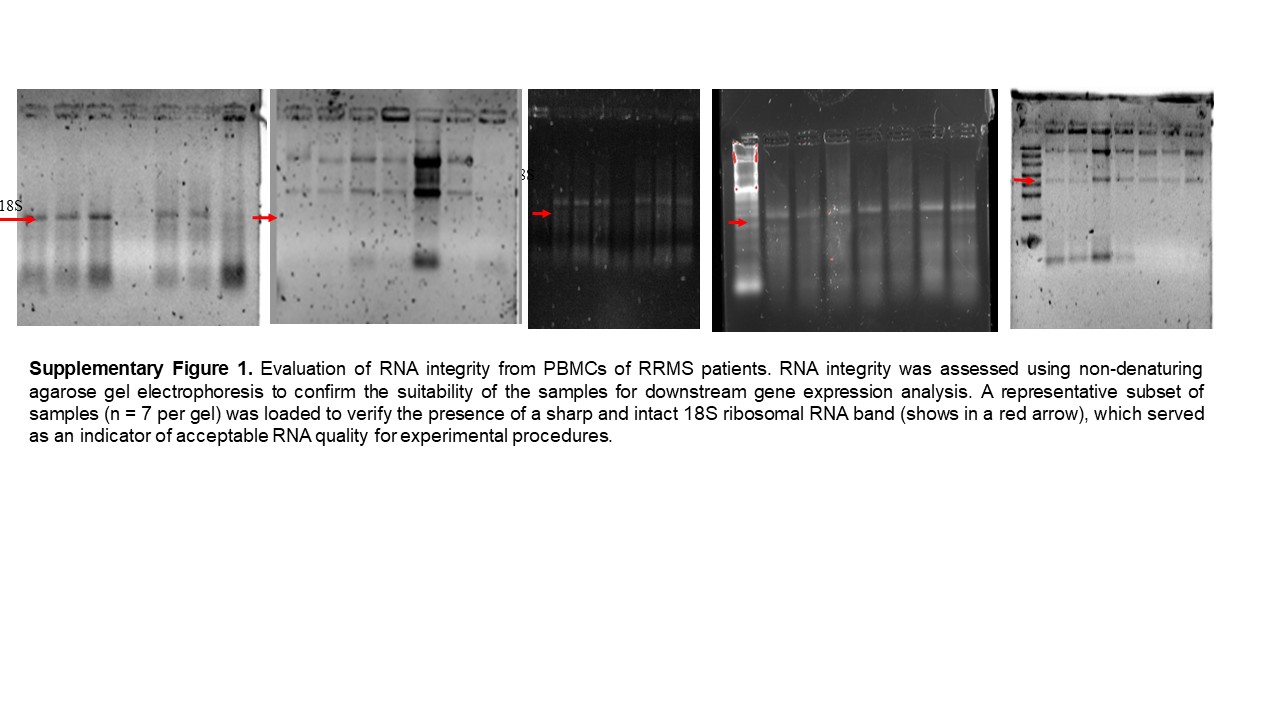

Supplement: Supplementary file 1 [file ijms-26-12094-s001.zip › Supp Figure 1.JPG]

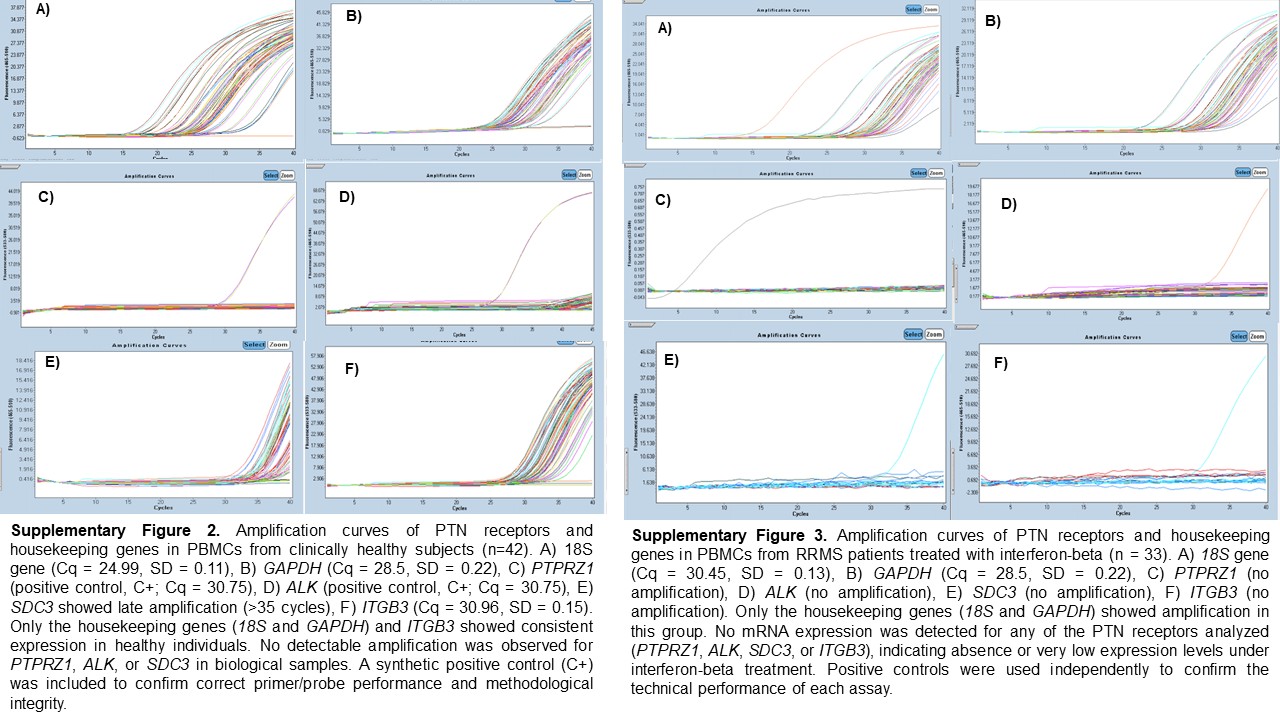

Supplement: Supplementary file 1 [file ijms-26-12094-s001.zip › Supp Figures 2 and 3.JPG]

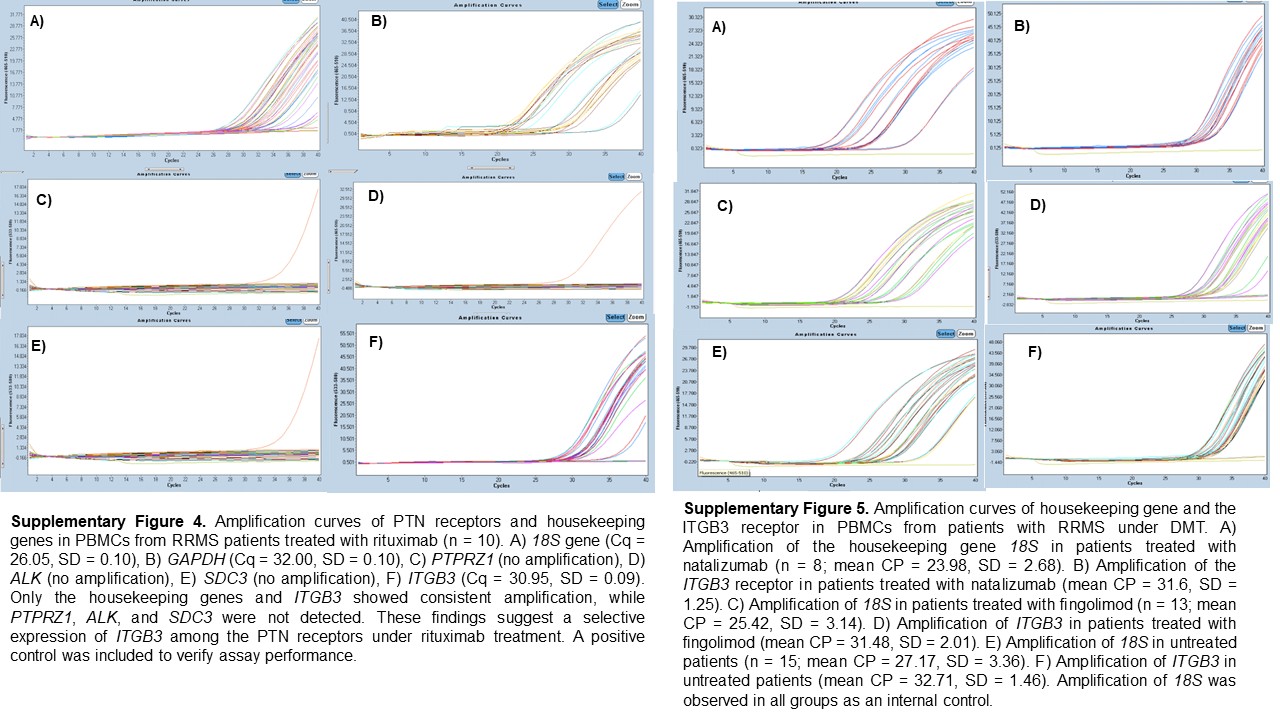

Supplement: Supplementary file 1 [file ijms-26-12094-s001.zip › Supp Figures 4 and 5.JPG]

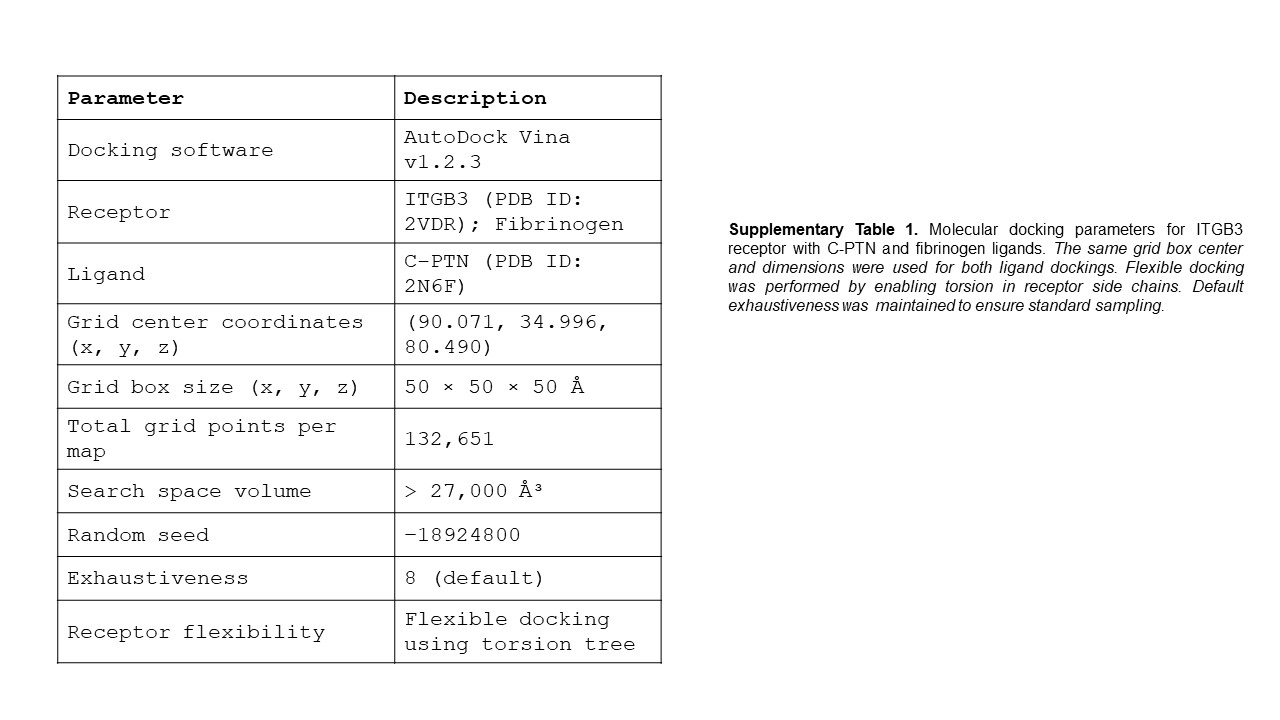

Supplement: Supplementary file 1 [file ijms-26-12094-s001.zip › Supp Table 1.JPG]

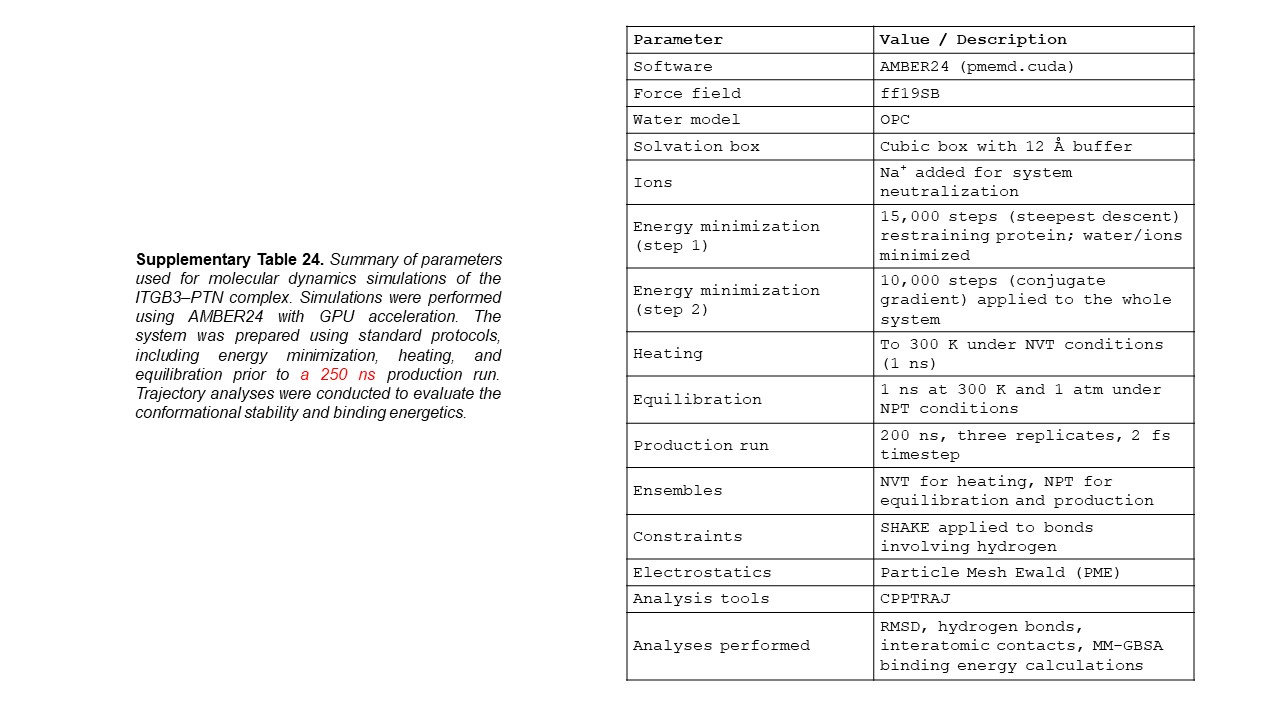

Supplement: Supplementary file 1 [file ijms-26-12094-s001.zip › Supp Table 4.JPG]
